# Tranilast for advanced heart failure in patients with muscular dystrophy: a single-arm, open-label, multicenter study

**DOI:** 10.1186/s13023-022-02352-3

**Published:** 2022-05-16

**Authors:** Tsuyoshi Matsumura, Hiroya Hashimoto, Masahiro Sekimizu, Akiko M. Saito, Yasufumi Motoyoshi, Akinori Nakamura, Satoshi Kuru, Takayasu Fukudome, Kazuhiko Segawa, Toshiaki Takahashi, Takuhisa Tamura, Tetsuo Komori, Chigusa Watanabe, Masanori Asakura, Koichi Kimura, Yuko Iwata

**Affiliations:** 1 Department of Neurology, National Hospital Organization Osaka Toneyama Medical Center, 5-1-1 Toneyama, Toyonaka, Osaka 560-8551 Japan; 2 Clinical Research Management Center, Nagoya City University Hospital, 1 Kawasumi Mizuho-Cho, Mizuho-Ku, Nagoya, Aichi 467-8602 Japan; 3 Clinical Research Center, National Hospital Organization Nagoya Medical Center, 4-1-1 Sannomaru, Naka-Ku, Nagoya, Aichi 460-0001 Japan; 4 Department of Neurology, National Hospital Organization Shimoshizu National Hospital, 934-5 Shikawatashi, Yotsukaido, Chiba, 284-0003 Japan; 5 Department of Clinical Research, National Hospital Organization Matsumoto Medical Center, 2-20-30 Muraimachi-Minami, Matsumoto, Nagano, 399-8701 Japan; 6 Department of Neurology, National Hospital Organization Suzuka Hospital, 3-2-1 Kasado, Suzuka, Mie 513-8501 Japan; 7 Department of Neurology, National Hospital Organization Nagasaki Kawatana Medical Center, 2005-1 Shimogumigo, Kawatana, Nagasaki, 859-3615 Japan; 8 Department of Cardiology, National Center of Neurology and Psychiatry, 4-1-1 Ogawahigashi, Kodaira, Tokyo 187-8551 Japan; 9 Department of Neurology, National Hospital Organization Sendai-Nishitaga Hospital, 2-11-11 Kagitorihoncho, Taihaku-ku, Sendai, Miyagi 982-8555 Japan; 10 Department of Intractable Diseases, National Hospital Organization Higashisaitama National Hospital, 4147 Kurohama, Hasuda, Saitama, 349-0196 Japan; 11 Department of Neurology, National Hospital Organization Hakone Hospital, 412 Kazamatsuri, Odawara, Kanagawa, 250-0032 Japan; 12 Department of Neurology, National Hospital Organization Hiroshima-Nishi Medical Center, 4-1-1 Kuha, Otake, Hiroshima 739-0696 Japan; 13 Department of Cardiovascular and Renal Medicine, Hyogo College of Medicine, 1-1 Mukogawa, Nishinomiya, Hyogo 663-8501 Japan; 14 Department of Laboratory Medicine/Cardiology, The Institute of Medical Science, The University of Tokyo, 4-6-1 Shirokanedai, Minato-ku, Tokyo, 108-8639 Japan; 15 Department of Cardiac Physiology, National Cerebral and Cardiovascular Center Research Institute, 6-1 Kishibe-Shimmachi, Suita, Osaka 564-8565 Japan

**Keywords:** Transient receptor potential cation channel subfamily V member 2, Muscular dystrophy, Heart failure, Brain natriuretic peptide, Tranilast

## Abstract

**Background:**

The transient receptor potential cation channel subfamily V member 2 (TRPV2) is a stretch-sensitive calcium channel. TRPV2 overexpression in the sarcolemma of skeletal and cardiac myocytes causes calcium influx into the cytoplasm, which triggers myocyte degeneration. In animal models of cardiomyopathy and muscular dystrophy (MD), TRPV2 inhibition was effective against heart failure and motor function. Our previous pilot study showed that tranilast, a TRPV2 inhibitor, reduced brain natriuretic peptide (BNP) levels in two MD patients with advanced heart failure. Thus, this single-arm, open-label, multicenter study aimed to evaluate the safety and efficacy of tranilast for heart failure.

**Methods:**

The study enrolled MD patients with advanced heart failure whose serum BNP levels were > 100 pg/mL despite receiving standard cardioprotective therapy. Tranilast was administered orally at 100 mg, thrice daily. The primary endpoint was the change in log (BNP) (Δlog [BNP]) at 6 months from baseline. The null hypothesis was determined based on a previous multicenter study of carvedilol results in a mean population Δlog (BNP) of 0.18. TRPV2 expression on peripheral blood mononuclear cell surface, cardiac events, total mortality, left ventricular fractional shortening, human atrial natriuretic peptide, cardiac troponin T, and creatine kinase, and pinch strength were also assessed.

**Results:**

Because of the poor general condition of many patients, only 18 of 34 patients were included and 13 patients could be treated according to the protocol throughout the 6-month period. However, there were no serious adverse events related to tranilast except diarrhea, a known adverse effect, and the drug was administered safely. TRPV2 expression on the mononuclear cell surface was elevated at baseline and reduced after treatment. Cardiac biomarkers such as BNP, human atrial natriuretic peptide, and fractional shortening remained stable, suggesting a protective effect against the progression of heart failure. In the per protocol set group, Δlog [BNP] was − 0.2 and significantly lower than that in the null hypothesis.

**Conclusions:**

Tranilast is safe and effective in inhibiting TRPV2 expression, even in MD patients with advanced heart failure. Further trials are needed to evaluate the efficacy of tranilast in preventing myocardial damage, heart failure, motor impairment, and respiratory failure.

*Clinical trial registration* The study was registered in the UMIN Clinical Trials Registry (UMIN-CTR: UMIN000031965, URL: http://www.umin.ac.jp/ctr/) [March 30, 2018] and the Japan Registry of Clinical Trials (jRCT, registration number: jRCTs031180038, URL: https://jrct.niph.go.jp/) [November 12, 2021]. Patient registration was started in December 19, 2018.

## Background

Advances in multidisciplinary medicine have greatly improved the prognosis of Duchenne muscular dystrophy (DMD) and other muscular dystrophies (MDs). Previously, there has been no effective treatment for MD other than steroids for DMD. However, the development of new therapies provides potential for an improved muscular function. In the past, respiratory failure and respiratory infections were the leading causes of death in MD, but advances in mechanical ventilation have markedly reduced respiratory death. Although widespread cardioprotective therapies, such as angiotensin-converting enzyme inhibitor (ACEI)/ angiotensin II receptor blocker (ARB) and beta-blockers, have led to improvements in heart failure, their benefits have been insufficient. Currently, death in MD is mainly caused by heart failure [1]. In addition, there is concern that improvement in motor function with new therapies may aggravate heart failure. Therefore, new options for the treatment of heart failure are urgently needed.

The transient receptor potential cation channel, subfamily V, member 2 (TRPV2) is a stretch-sensitive calcium channel [2, 3]. It is usually localized in the intracellular membrane compartments but translocates to the cytoplasmic membrane in damaged myocytes or cardiomyocytes. In the cytoplasmic membrane, TRPV2 enhances the influx of calcium and triggers cell damage. TRPV2 overexpression in the cytoplasmic membrane has been observed in the cardiac and skeletal muscles of animal models of MDs [3, 4]. TRPV2 overexpression in the sarcolemma has also been observed in the skeletal muscle and cardiomyocytes of patients with MD [3, 4] and in the cardiomyocytes of patients with dilated cardiomyopathy (DCM) [4].

Transgenic mice with cardiac-specific TRPV2 overexpression develop DCM [3]. TRPV2 inhibition also ameliorated the severity of muscle pathology, motor function, and cardiac function in various animal models of MDs and DCM [4–7]. We previously developed a high-throughput screening method and detected some compounds that inhibited TRPV2 [4, 8]. Tranilast, which has already been approved as a small molecule anti-allergic drug, is one of such compounds. Tranilast was effective in hamster models of cardiomyopathy and myocyte degeneration by resulting in the effective removal of TRPV2 from the sarcolemma of DCM hamsters [4, 5, 9]. Importantly, we performed a pilot study in which tranilast was used to treat two patients with MD and advanced cardiomyopathy. In both patients, the serum level of brain natriuretic peptide (BNP) decreased after tranilast therapy [10]. These patients also showed an improvement in echocardiographic findings after more than 1 year of treatment [11].

Based on these findings, we subsequently conducted a single-arm, open-label, multicenter study of tranilast as an additional therapy in MD patients with heart failure, and BNP levels were > 100 pg/mL during standard cardiac protection therapy [12]. BNP is a standard cardiac function indicator that is highly correlated with heart failure severity and prognosis. In MD, BNP levels ≥ 100 pg/mL is considered to be a predictive risk factor for death [13].

## Results

### Subject characteristics

**Fig. 1:**
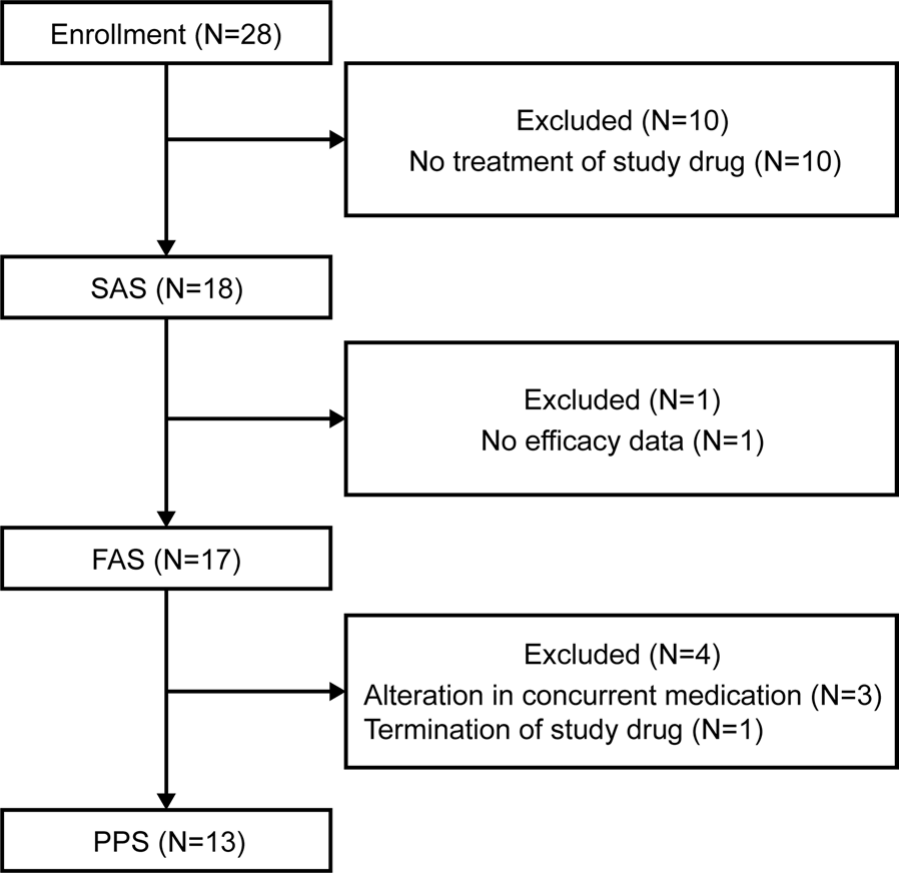
Patient inclusion flowchart

Among the 34 patients initially screened, 28 patients were enrolled. Of them, only 18 patients could take the tranilast (safety analysis set: SAS) (Fig. 1). The reasons for drop out were severe mitral regurgitation in three patients, lethal arrhythmia in two patients, cerebral vascular disorders in two patients, contraindication in two patients, and refusal to join the study after the coronavirus disease 2019 (COVID-19) pandemic in one patient. Although the target number of patients was not achieved, recruitment was terminated at the end of August 2020 because of difficulties in enrolling patients after the COVID-19 pandemic.

Among the 18 treated patients, one patient stopped medication due to severe anorexia 17 days after the initiation of tranilast. This case was excluded from the full analysis set (FAS) because no follow-up data could be obtained. In addition, three patients had altered cardiac drugs regimen (dosage change), and one patient terminated tranilast due to recurrent diarrhea. Thus, the number of per protocol set (PPS) was 13 (Fig. 1). The FAS consisted of 13 DMD, 3 BMD, and 1 LGMD patients. Fifteen patients were diagnosed genetically or immunologically, whereas one DMD and one LGMD patient were diagnosed pathologically. The FAS profiles are presented in Table 1.

**Table 1:** Patient characteristics

Items		FAS (n = 17)
Age (years)	Mean ± SD	33.6 ± 8.0
Sex	Male/female	16 (94.1%)/1 (5.9%)
Diseases	Duchenne muscular dystrophy	13 (76.5%)
	Becker muscular dystrophy	3 (17.6%)
	Limb-Girdle muscular dystrophy	1 (5.9%)
Previous illness	No/Yes	11 (64.7%)/6 (35.3%)
	Severe heart failure	3 (17.6%)
	Lethal arrhythmia	2 (11.8%)
	acute nephritis/severe renal dysfunction	1 (5.9%)
	Thrombosis/embolism	1 (5.9%)
Complications	No/yes	12 (70.6%)/5 (29.4%)
	Biliary disorders (gall stone, cholecystolithiasis)	1 (5.9%)
	Rash	2 (11.8%)
	Others	2 (11.8%)
Allergy	No/yes	9 (52.9%)/8 (47.1%)
	Rhinitis	3 (17.6%)
	Hay fever	2 (11.8%)
	Atopic dermatitis	3 (17.6%)
	Drug hypersensitivity (Not for tranilast)	2 (11.8%)
Mitral regurgitation	Absent or trivial/mild/moderate	7 (41.2%)/7 (41.2%)/3 (17.6%)
BNP (pg/mL)	GM ± GSD IQR	192.9 ± 1.72 (132–260)
hANP (pg/mL)	GM ± GSD IQR	189.7 ± 1.74 (152–284)
cTnT (ng/mL)	GM ± GSD IQR	0.026 ± 1.76 (0.02–0.03)
FS (%)	Mean ± SD IQR	9.0 ± 3.8 (6–13)
LVDD (mm)	Mean ± SD IQR	58.1 ± 6.3 (55–61)
Motor function	Ambulant/non-ambulant	1 (5.9%)/ 16 (94.1%)
Mechanical ventilation	No/intermittent/full-time	2 (11.8%)/13 (76.5%)/ 2 (11.8%)
Oxygen inhalation	No/yes	16 (94.1%)/1 (5.9%)
Nutrition	Oral/tube feeding	8 (47.1%)/9 (52.9%)
ACEI/ARB	Yes/no	15 (88.2%)/2 (11.8%)
Beta-blocker	Yes/no	15 (88.2%)/2 (11.8%)
Digitalis	Yes/no	2 (11.8%)/15 (88.2%)
Diuretics (except aldosterone receptor antagonist)	Yes/no	6 (35.3%)/11 (64.7%)
Aldosterone receptor antagonist	Yes/no	8 (47.1%)/9 (52.9%)
Cardiotonic agents	Yes/no	3 (17.6%)/14 (82.4%)
Anti-arrhythmic agents	Yes/no	10 (58.8%)/7 (41.2%)

^*^The diagnosis was based on genetic analysis in 15 patients and muscle biopsy in one DMD and one LGMD patient

FAS, full analysis set; GM, geometric mean; GS, geometric standard deviation; IQR, inter quartile range; BNP, brain natriuretic peptide; hANP, human atrial; cTnT, cardiac troponin T; FS, fractional shortening; LVDD; Left Ventricular Diastolic Dysfunction; ACEI/ARB, angiotensin-converting enzyme inhibitors/ angiotensin-receptor blockers

### Primary endpoint

**Fig. 2:**
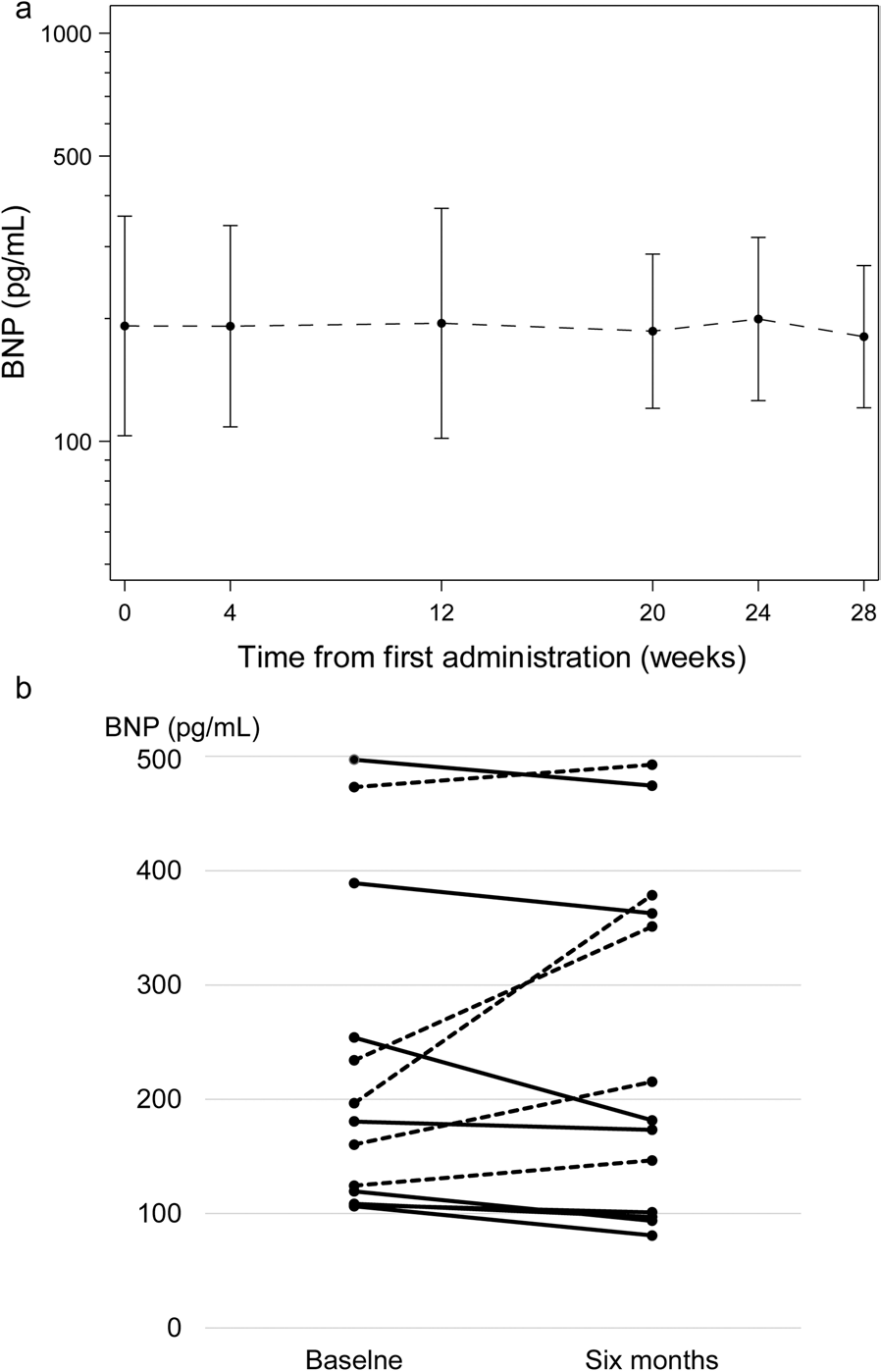
Brain natriuretic peptide (BNP) results. **a** Longitudinal changes, error bar: 95% confidence interval. **b** Average individual changes at baseline and 6 months after treatment. Solid line: patients with decrease in BNP; dash line: patients with increase in BNP

Although there was variation in the BNP level of individual patients, as participants in this study had advanced heart failure and poor general condition, the mean value remained stable (Fig. 2a). In the PPS group, 8 and 5 patients had lower and higher BNP levels 6 months after treatment, respectively (Fig. 2b). The geometric mean ± geometric standard deviation of serum BNP level of the PPS was 191.7 ± 1.71 pg/mL at baseline and 191.3 ± 1.88 pg/mL 6 months after treatment (Table 2). The percent change in BNP was − 0.2% (95% CI − 15.7 to 18.1) and significantly lower than that in the null hypothesis (*p* = 0.036).

**Table 2:** Comparison of data between baseline and 6 months after or at the cessation of the treatment

	Baseline	After treatment	Change (95% CI)	*p*
	(GM ± GSD)	(GM ± GSD)	Percentage change	
*BNP (pg/mL)*				
FAS	192.9 ± 1.72	188.8 ± 1.92	− 2.1% (− 21.5 to 22.0)	0.071
PPS	191.7 ± 1.71	191.3 ± 1.88	− 0.2% (− 15.7 to 18.1)	0.036
hANP (pg/mL)	189.7 ± 1.74,	199.1 ± 2.19	5.0% (− 13.6 to 27.6)	0.605
cTnT (mg/dL)	0.026 ± 1.76	0.030 ± 1.88	14.6% (1.1 to 29.9)	0.035
CK (IU/dL)	269 ± 2.12	278 ± 2.41	3.5% (− 23.0 to 39.2)	0.808
	(mean ± SD)	(mean ± SD)	Change	
FS (%)	9.02 ± 3.83%	9.24 ± 4.06%	0.22 (− 0.2 to 0.8)	0.401
Pinch (kg)	1.00 ± 1.74	0.78 ± 1.28	− 0.29 (− 0.83 to 0.25)	0.269
*MDQoL-60*				
Mental stability	52.6 ± 16.1	55.8 ± 22.6	2.5 (− 5.7 to 10.7)	0.523
ADL	49.5 ± 29.8	53.6 ± 30.3	− 0.8 (− 11.0 to 9.3)	0.862
Environment	71.6 ± 17.1	71.5 ± 14.1	− 0.9 (− 7.3 to 5.4)	0.763
Hope	61.8 ± 22.7	55.5 ± 18.9	− 6.2 (− 19.7 to 7.3)	0.343
Activity	42.4 ± 30.4	51.3 ± 28.1	7.0 (− 11.9 to 25.9)	0.441
Sense of well-being	63.2 ± 31.6	58.9 ± 25.3	− 8.3 (− 26.6 to 9.9)	0.344
Human relation	63.2 ± 21.4	58.3 ± 23.4	− 5.7 (− 16.9 to 5.5)	0.297
Family	72.6 ± 21.1	73.0 ± 20.6	1.0 (− 6.4 to 8.4)	0.777
Sex	64.2 ± 28.4	72.2 ± 20.3	9.4 (− 0.1 to 19.0)	0.052
Respiration and bulbar function	58.5 ± 18.8	61.7 ± 31.0	0.0 (− 13.7 to 13.7)	1.000
Defecation	64.7 ± 24.7	65.0 ± 35.4	0.8 (− 16.2 to 17.9)	0.918
*SF-12*				
Physical function	9.0 ± 14.1	5.6 ± 5.6	− 4.7 (− 14.5 to 5.0)	0.315
Role physical	26.1 ± 18.9	29.8 ± 18.8	0.9 (− 11.0 to 12.7)	0.880
Bodily pain	49.4 ± 11.7	48.4 ± 12.1	− 3.7 (− 10.2 to 2.7)	0.238
General health	51.6 ± 5.3	54.7 ± 7.7	3.1 (− 2.2 to 8.4)	0.234
Vitality	42.2 ± 11.1	45.1 ± 8.7	2.4 (− 3.7 to 8.6)	0.413
Social function	46.5 ± 12.1	43.6 ± 16.1	− 5.3 (− 13.9 to 3.3)	0.204
Role emotional	49.5 ± 9.3	38.9 ± 14.8	− 9.7 (− 17.5 to − 1.9)	0.018
Mental health	51.4 ± 6.9	47.8 ± 11.5	− 3.6 (− 9.6 to 2.4)	0.219

GM, geometric mean; GS, geometric standard deviation; CI, confidence interval, BNP, brain natriuretic peptide; FAS, full analysis set; PPS, per protocol set; hANP, human atrial; cTnT, cardiac troponin T; CK, creatinine kinase; FS, fractional shortening; MDQoL-60, Muscular Dystrophy Quality of life-60; ADL, activities of daily living

*Percentage change = (geometric mean ratio − 1) × 100

As the null hypothesis was derived from the carvedilol trial in DMD patients, we also assessed the primary endpoint in DMD patients alone: in 10 DMD patients in the PPS, serum BNP level was 202.1 ± 1.81 pg/mL at baseline and 188.2 ± 1.93 pg/mL at 6 months after treatment. The percent change in BNP was − 6.9% (95% CI − 21.2 to 7.6) and significantly lower than that in the null hypothesis (*p* = 0.007).

## Secondary endpoints

### TRPV2 expression in peripheral blood mononuclear cell surfaces

The ratio of mononuclear cells expressing TRPV2 on the cell surface was elevated at baseline 30.67 ± 10.11% and significantly reduced at 4 weeks after treatment (9.91 ± 9.51%) (p < 0.001). There was a weak positive correlation trend between TRPV2 expression and log (BNP); however, no significant differences were observed (Pearson correlation coefficient r = 0.286, *p* = 0.222).

### Other assessments

Among the cardiac indices, fractional shortening (FS) (*p* = 0.401) and human atrial natriuretic peptide (hANP) (*p* = 0.605) were stable. However, cardiac troponin T (cTnT) levels were mildly elevated 6 months after or at the cessation of treatment (*p* = 0.035) (Table 2). There were no significant changes in creatine kinase (CK) (*p* = 0.808); pinch strength (*p* = 0.269); and all categories of Muscular Dystrophy Quality of Life-60 (MDQoL-60) and Short Form 12 Health Survey (SF-12), except for emotional role in SF-12 (*p* = 0.018).

### Cardiac events and total mortality

**Fig. 3:**
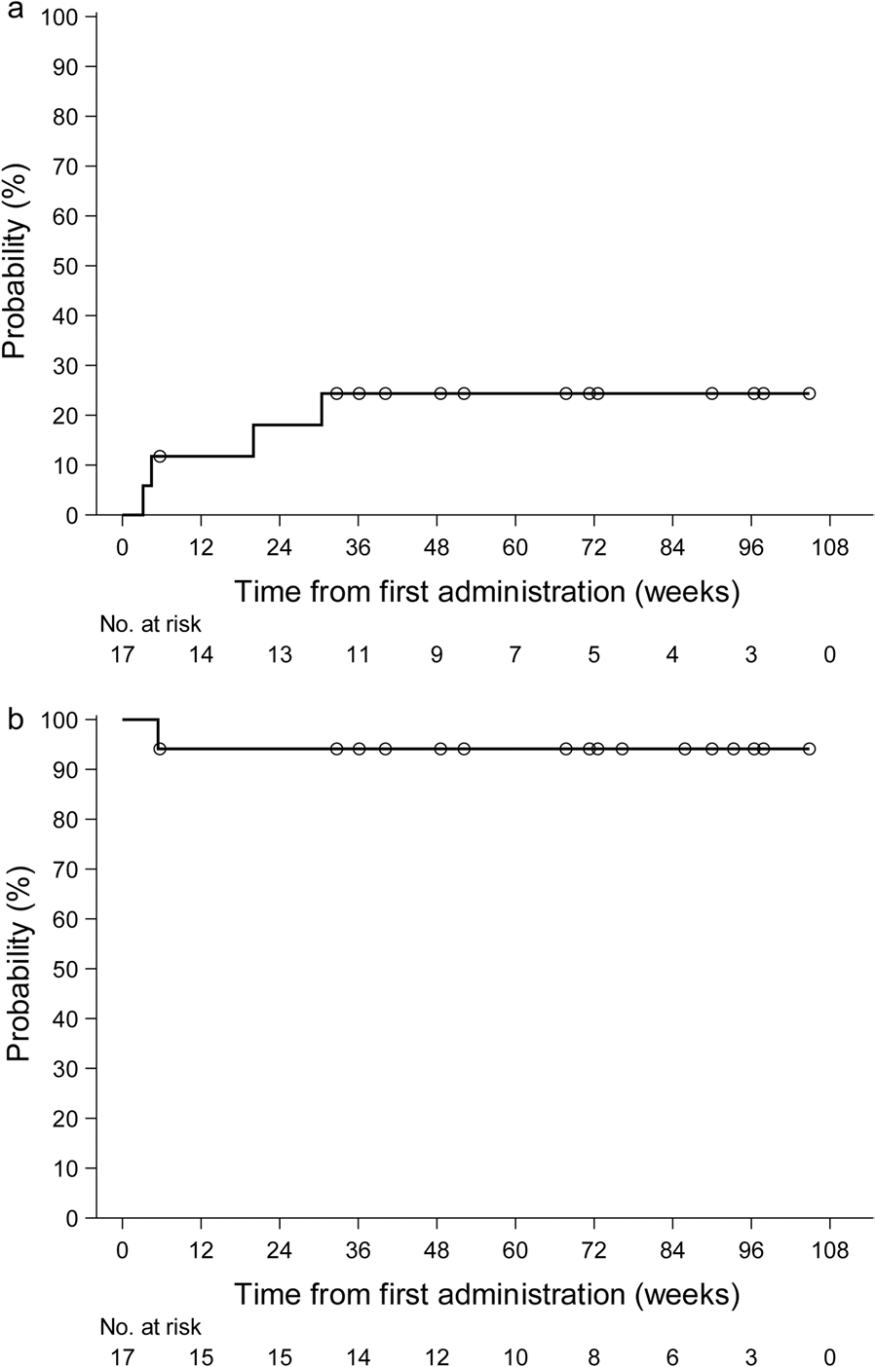
Cumulative incidence of cardiac event and survival curve. **a** Cumulative incidence of cardiac event: the probability of a cardiac event is 18.1% at 24 weeks. **b** Survival curve: the survival rate is 94.1% at 24 weeks

Four unexpected events occurred during the study period. The first patient had chronic pneumonia before the initiation of tranilast. He developed sepsis and died 67 days after treatment. The Safety Monitoring Committee (SMC) judged this event as a severe adverse event (SAE) not related to tranilast. In the second patient, their home doctor changed the eplerenone dose at 7 months after treatment because of miscommunication. In this case, the dose change was not based on worsening of cardiac function. However, we treated it as a cardiac event because the changed dose was maintained. The third patient discontinued the digitalis due to atrioventricular block 3 weeks after treatment. The fourth patient was admitted to the hospital because of recurrent diarrhea and dehydration 5 months after treatment. Given that diarrhea is a known side effect of tranilast, the SMC judged this event as an SAE related to tranilast. The 24-week cumulative incidence of cardiac events was 18.1% (Fig. 3a), while the survival rate was 94.1% (Fig. 3b).

### SAE and other adverse events

**Fig. 4:**
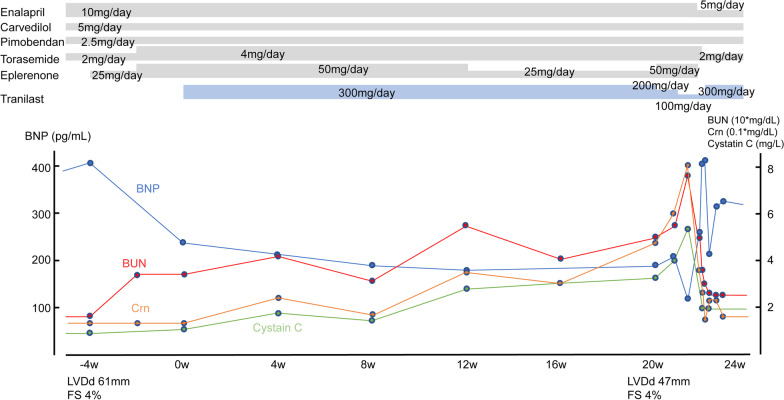
Clinical course of the patients who showed renal dysfunction. The patient had been treated with standard cardioprotective therapy, including ACEI and beta-blocker, but continued to have elevated BNP and enlarged LVDD. Because of nocturnal bradycardia, carvedilol dose escalation was not feasible. When BNP exceeded 400 pg/mL and LVDD became 61 mm, we considered induction of tranilast. Before introducing tranilast, we started eplerenone and increased the dose of torasemide. Then patient's BNP decreased to less than 300 pg/mL at the start of tranilast. After initiation of tranilast, his BNP decreased, and renal indices deteriorated gradually. Firstly, we reduced eplerenone, but the renal function worsened further. Then tranilast was reduced. However, renal function worsened rapidly. In addition, the LVDD was significantly reduced to 47 mm. From these facts, we suspected hypovolemia by diuretics and renal dysfunction due to ACEI. Reduction of ACEI and diuretics resulted in rapid improvement in renal function and an increase in BNP. After re-introducing tranilast, renal indices and BNP became stable. ACEI, angiotensin-converting enzyme inhibitors; BNP, brain natriuretic peptide; LVDD, Left Ventricular Diastolic Dysfunction

Three SAEs occurred in three patients within 6 months of the study. Of these, two were cardiac events in two patients. The other patient showed improvement in cardiac indices (BNP, hANP, and LVDD). However, renal indices (blood urea nitrogen (BUN), creatinine (Crn), and cystatin C) worsened. A decrease in eplerenone or tranilast dosage did not improve renal function. We considered that hypovolemia and ACEI were associated with this event because we induced eplerenone and increased torasemide before the initiation of tranilast. After discontinuing eplerenone and decreasing torasemide and ACEI, cystatin C and Crn levels recovered within a few days. We re-introduced tranilast because BNP levels increased after a decrease in tranilast. As a result, BNP levels decreased and renal function remained stable (Case 16 in Fig. 4). The SMC judged this event as an SAE unrelated to tranilast.

Chronic kidney disease, an increase in cystatin C, and a decrease in white blood cells (WBC) were reported in one subject, respectively.

For laboratory data, the mean ± standard deviation (SD) of cystatin C, Crn, BUN, UA, and WBC at baseline versus after 24 weeks or at cessation of the treatment were 0.863 ± 0.188 mg/L vs 1.039 ± 0.413 mg/L (*p* = 0.090), 0.14 ± 0.10 mg/dL vs 0.19 ± 0.20 mg/dL (*p* = 0.234), 14.3 ± 6.9 mg/dL vs 19.1 ± 13.9 mg/dL (*p* = 0.152), 5.1 ± 1.2 md/dL vs 3.4 ± 1.5 md/dL (p < 0.001), and 5536 ± 1043/μL vs 6452 ± 2533/μL (*p* = 0.101), respectively.

In the previous pilot study, one patient showed a transient increase in pulse rate and premature ventricular contraction (PVC) [10]. In this study, five patients showed an increase in PVC of more than 15%, and three patients showed novel triplet or quadruplet on Holter electrocardiogram (ECG). However, all these patients were asymptomatic, and no new lethal arrhythmias were detected. The mean ± SD heart rate and number of PVC at baseline versus 24 weeks were 77.8 ± 10.6 bpm vs. 78.6 ± 9.0 bpm (*p* = 0.524) and 786.7 ± 1384.3 beats/day vs. 1588.3 ± 2710.5 beats/day (*p* = 0.065), respectively.

### Other biomarkers

Eosinophil levels were transiently increased at 4 weeks (4.1 ± 3.4% at baseline, 6.2 ± 6.2%: *p* = 0.037) but recovered spontaneously. Post-treatment, hemoglobin (Hb) levels and RBC counts were mildly reduced after treatment (Hb: 13.9 ± 1.3 g/dL at baseline versus 12.7 ± 1.4 g/dL at 24 weeks or cessation of the treatment, p < 0.001; RBC: 445*10^4^ ± 38*10^4^/μL at baseline versus 395*10^4^ ± 43*10^4^/μL at 24 weeks or cessation of the treatment, p < 0.001). Meanwhile, there were no apparent changes in liver function, electrolytes, or urinary tests. Atrioventricular block (AVB) was not observed in the ECG, but bundle branch blocks were found in six patients. Ischemic changes were observed in two patients at baseline. One patient showed AVB, as described for cardiac events. Only five patients underwent spirometry. The mean ± SD of %FVC and FEV1% at baseline versus 24 weeks were 54.6 ± 40.4% vs. 51.9 ± 40.8% (*p* = 0.238) and 87.4 ± 20.0% vs. 87.4 ± 12.8% (*p* = 0.994), respectively. Chest radiography showed congested lung and pleural effusion in three and two cases at baseline, two and one case at 12 weeks, and one each at 24 weeks, respectively.

## Discussion

To the best of our knowledge, this was the first-in-human trial for heart failure, excluding our pilot study. The study population consisted of MD patients with advanced heart failure who had an inadequate response to standard treatment, but there were no serious safety issues. Analysis of TRPV2 expression at the surface of peripheral mononuclear cells confirmed that tranilast had a TRPV2 inhibitory effect even in humans. Although no obvious improvement in cardiac function was observed, preventive effects of worsening were suggested.

There were several difficulties and limitations in this study. MD patients have a significantly reduced cardiac load due to reduced mobility and mechanical ventilator use, and younger age (< 50 years of age) with low risk of arteriosclerosis and hypertension. As such, cardiac biomarkers including BNP are often within normal values until the advanced stage of heart failure. Therefore, we enrolled only subjects with advanced heart failure (BNP > 100 pg/mL) and unstable general condition. This made it challenging to recruit patients. Although we screened 36 patients during the 20-month enrollment period, only 18 patients could start treatment. The target population was 20 patients, but we decided to terminate enrollment with the addition of difficulty in seeing patients during the COVID-19 pandemic.

Although tranilast has been on the market for more than 35 years, there are no clinical data for its use in patients with severe heart failure, except for a pilot study [10]. Animal studies have shown that tranilast has a dose-dependent effect on heart failure. However, previous clinical trials using a dose of 600 mg/day for the prevention of post-percutaneous transluminal coronary angioplasty restenosis found increased liver dysfunction and cystitis-like symptoms [14, 15]. Therefore, we set the tranilast dose to 300 mg/day, which is accepted by medical insurance. Consequently, there were no changes in liver function and no complaints of cystitis-like symptoms in the current study.

Three SAEs have occurred from the study initiation till the time of submission of this manuscript, and one of these resulted in death. However, this was not judged to be related to tranilast. One case had increased diuretics use before initiation of tranilast, which caused hypovolemia and renal dysfunction. Only one case of recurrent diarrhea, a known side effect of tranilast, was considered to be related to the treatment. Further, there were two other cardiac events that involved alterations in cardiac medication. In the pilot study, we observed deterioration in renal function and an increase in PVC and pulse. Thus, these were checked in the current study. BUN and Crn were unchanged; cystatin C was increased; and UA, RBC, and Hb levels decreased. Although the effect of tranilast cannot be ruled out, we should consider the effects and interaction of ACEI and diuretics, which were taken by many study subjects. With respect to arrhythmia, some patients showed an increase in PVC, but all of them were asymptomatic. There were no significant changes in the number of PVCs or pulse rate before and after treatment.

In the advanced stage of MD, swallowing function is also impaired; therefore, safety in oral administration is also a challenge. In this study, no medication-related problems were reported because they could choose the dosage form freely. Collectively, these results confirm that tranilast is safe even for MD patients with advanced heart failure. Given that this study was a single-arm, open-label study, we did not have a control group. However, of the 16 patients who could not start the study treatment, 2 patients received tranilast outside the study, 5 patients survived, 4 patients died, and 5 patients had an unknown outcome. It is not possible to compare them because they were not observed under the same conditions. Nonetheless, there is a possibility that tranilast may improve cardiac events and mortality.

To investigate the inhibitory effect of tranilast on TRPV2, we assessed the expression of TRPV2 on the surface of peripheral blood mononuclear cells, as it was not possible to search the myocardium. TRPV2 expression was apparently decreased post-treatment, supporting the pharmacological effect; TRPV2 inhibition induced the removal of TRPV2 cytoplasmic membrane retention, as reported previously [10]. Although there was no obvious correlation between TRPV2 expression and log (BNP), we could consider several reasons for it. First, in chronic diseases such as MD, the accumulation of myocardial degeneration leads to cardiac dysfunction, making it difficult to assess the correlation in real-time. Second, other than myocardial degeneration, volume load, vascular resistance, and blood pressure also affect BNP. Third, since the participants of this study were advanced cases, instability of general conditions could also impact the BNP.

Although the participants in this study had severe heart failure and poor health conditions, cardiac indices such as BNP, hANP, and FS were stable during the 6 months of treatment. In addition, although the difference was not remarkable, the 6-month BNP was lower than that at baseline in more than half of the patients. Consequently, the null hypothesis for the primary endpoint of Δlog [BNP] is rejected in the PPS. We expected that TRPV2 inhibition would improve myocardial degeneration, which would be expressed as a decrease in cTnT levels. However, the cTnT levels were increased, to a certain extent, after treatment. This was mainly due to one patient dropping out because of recurrent diarrhea and another patient with a relatively high and fluctuating cTnT. However, there was no apparent decrease in other cases. In our previous study, cardiac troponin levels peaked before the decline in cardiac function [16]. To our best knowledge, this study is the first prospective intervention trial for patients with heart failure. We included only patients who did not respond sufficiently to standard cardioprotective therapy. Further trials targeting patients with early-stage heart failure are needed to evaluate the effects of tranilast in improving myocardial degeneration and preventing heart failure.

TRPV2 inhibition is also expected to be effective for the management of skeletal muscle diseases. Thus, we assessed its effects on the skeletal muscle according to CK, pinch strength, and respiratory function. However, we could not detect significant changes in these parameters, particularly because the participants had advanced disease. As such, data from patients with earlier stage disease are needed. No remarkable changes were observed in the MDQoL-60 and SF-12 scores. There was a trend toward worse scores on the items of “role emotional,” “social functioning,” and “mental health” of the SF-12 and on the items of “hope,” “sense of well-being,” and “human relation” of the MDQoL-60. However, it was considered that the COVID-19 pandemic may have had a more significant influence on these results than did this study [17].

This study has some limitations. Firstly, we could not use high dose of tranilast from safety concerns. In animal models, the effect of tranilast was dose-dependent. However, we had to fix the dose to 300 mg/day, as approved by medical insurance, because side effects were frequent in past clinical trials using 600 mg/day [14, 15]. Secondly, 6 months may not be sufficient to assess the effect of tranilast on cardiac function. In the pilot study, changes in echocardiographic findings were observed after more than one year. Because this study allowed an extension of the treatment period of 116 weeks for those who wished to do so, we must await the data from the extension study. Thirdly, due to the lack of data on tranilast for heart failure, the subjects were limited to patients whose BNP levels were > 100 pg/mL during standard treatment. It made it difficult for patient recruitment and evaluation of the effect on skeletal muscle. It is assumed that tranilast is most effective when myocardial damage is active, early stage of heart failure. Indeed, in DMD, cardiac troponin showed a peak in the teenage years and preceded cardiac dysfunction [16]. Because we confirmed the safety of tranilast even in patients with heart failure, it is necessary to conduct a double-blind study in earlier patients to evaluate its efficacy in myocardial and skeletal muscle damage.

## Conclusions

Tranilast is safe and effective in inhibiting TRPV2 expression, even in MD patients with advanced heart failure. Although no noticeable improvement in cardiac function was observed, tranilast may have the potential to prevent the progression of cardiac dysfunction and reduce cardiac events and mortality. Clinical trials targeting patients with earlier stage heart failure are needed to evaluate the effects of tranilast, including on the skeletal muscle.

## Methods

This single-arm, open-label, multicenter study aimed to evaluate the safety and effects of tranilast for heart failure in patients with MD. Details of the study protocol have been reported previously [12]. The subjects were MD patients with advanced heart failure. The inclusion criteria were (1) age > 13 years, (2) serum BNP levels > 100 pg/mL at time of enrollment, (3) receiving standard cardioprotective therapy, (4) without acute clinical status and severe arrhythmia, and (5) renal/liver dysfunction leukopenia. Tranilast was administered orally at 100 mg thrice daily for 28 weeks. The dosage form was freely selectable from capsules, fine granules, and dry syrup according to the swallowing function and patient preference. Patients will continue to receive tranilast for 116 weeks if they provided consent at 28 weeks. Based on the pilot study [10], a multicenter trial of carvedilol [18] and consideration of drop-outs, we set the target number of subjects to 20.

The primary endpoint of this study was the change in log(BNP) (Δlog [BNP]) for 6 months (mean data at 20, 24, and 28 weeks) from baseline. For ease of interpretation, the results were presented as the percent change. The null hypothesis was determined based on a previous multicenter study of carvedilol results in a mean population Δlog [BNP] of 0.18, which corresponds to 19.7% in the percent change [12, 18]. The secondary endpoints were (1) cardiac events; (2) total mortality; (3) left ventricular FS; (3) hANP and (cTnT) levels, (4) TRPV2 expression on peripheral blood mononuclear cell surfaces, (5) pinch strength, (6) CK level, (7) MDQoL-60 [19] and the SF-12, and adverse events.

The analyses of the TRPV2 expression in mononuclear cells (MNCs) were performed largely as previously reported [10]. BD Phosflow™ Lyse/Fix Buffer was used to prepare the peripheral blood MNCs from blood samples. Prepared MNCs were immobilized on glass slides, permeabilized with 0.1% Triton™ X-100 (Axis-SHIELD PoC AS), incubated with the affinity purified rabbit anti-human TRPV2 antibody (1: 100 dilution) overnight at 4℃, and then incubated for 30 min at room temperature with Alexa Fluor 488 goat anti-rabbit IgG (H + L) (1: 500 dilution) (Invitrogen, Carlsbad, USA). Images of MNCs stained with TRPV2 antibody were obtained through confocal laser scanning microscopy (FLUOVIEWTM FV3000, Olympus, Tokyo, Japan) mounted on an objective lens (Olympus) and analyzed (total number of MNCs analyzed: > 100) using the National Institute of Health Imaging software program.

Efficacy analysis was performed in the FAS, with additional analysis in the PPS to assess the sensitivity of the primary endpoint. A safety analysis was performed in the SAS that received at least one dose of tranilast. Patient data collection and central monitoring were achieved using an electronic system. A scheduled monitoring report on the progress of the study was issued once per year. The primary and secondary endpoints and safety were analyzed when all patients completed the 6-month assessment.

## Data Availability

The datasets used and/or analyzed during the current study are available from the corresponding author on reasonable request.

## Abbreviations

ACEI: Angiotensin-converting enzyme inhibitor
ARB: Angiotensin II receptor blocker
AVB: Atrioventricular block
BNP: Brain natriuretic peptide
BUN: Blood urea nitrogen
CK: Creatine kinase
COVID-19: Coronavirus disease 2019
Crn: Creatinine
cTnT: Cardiac troponin T
DCM: Dilated cardiomyopathy
DMD: Duchenne muscular dystrophy
ECG: Electrocardiogram
FAS: Full analysis set
FS: Fractional shortening
hANP: Human atrial natriuretic peptide
Hb: Hemoglobin
LVDD: Left Ventricular Diastolic Dysfunction
MD: Muscular dystrophy
MDQoL-60: Muscular Dystrophy Quality of life-60
MDs: Muscular dystrophies
PPS: Per protocol set
PVC: Premature ventricular contraction
RBC: Red blood cell
SAE: Severe adverse event
SAS: Safety analysis set
SD: Standard deviation
SMC: Safe Monitoring Committee
TRPV2: Transient receptor potential cation channel subfamily V member 2
UA: Uric acid
WBC: White blood cells

## References

[1] Matsumura T, Saito T, Fujimura H, Shinno S, Sakoda S (2011). A longitudinal cause-of-death analysis of patients with Duchenne muscular dystrophy. Rinsho Shinkeigaku.

[2] Muraki K, Iwata Y, Katanosaka Y, Ito T, Ohya S, Shigekawa M (2003). TRPV2 is a component of osmotically sensitive cation channels in murine aortic myocytes. Circ Res.

[3] Iwata Y, Katanosaka Y, Arai Y, Komamura K, Miyatake K, Shigekawa M (2003). A novel mechanism of myocyte degeneration involving the Ca2+-permeable growth factor—regulated channel. J Cell Biol.

[4] Iwata Y, Ohtake H, Suzuki O, Matsuda J, Komamura K, Wakabayashi S (2013). Blockade of sarcolemmal TRPV2 accumulation inhibits progression of dilated cardiomyopathy. Cardiovasc Res.

[5] Iwata Y, Katanosaka Y, Shijun Z, Kobayashi Y, Hanada H, Shigekawa M (2005). Protective effects of Ca2+ handling drugs against abnormal Ca2+ homeostasis and cell damage in myopathic skeletal muscle cells. Biochem Pharmacol.

[6] Iwata Y, Katanosaka Y, Arai Y, Shigekawa M, Wakabayashi S (2009). Dominant-negative inhibition of Ca2+ influx via TRPV2 ameliorates muscular dystrophy in animal models. Hum Mol Genet.

[7] Zanou N, Iwata Y, Schakman O, Lebacq J, Wakabayashi S, Gailly P (2009). Essential role of TRPV2 ion channel in the sensitivity of dystrophic muscle to eccentric contractions. FEBS Lett.

[8] Iwata Y, Katayama Y, Okuno Y, Wakabayashi S (2018). Novel inhibitor candidates of TRPV2 prevent damage of dystrophic myocytes and ameliorate against dilated cardiomyopathy in a hamster model. Oncotarget.

[9] Iwata Y, Wakabayashi S, Ito S, Kitakaze M (2020). Production of TRPV2-targeting functional antibody ameliorating dilated cardiomyopathy and muscular dystrophy in animal models. Lab Investig.

[10] Matsumura T, Matsui M, Iwata Y, Asakura M, Saito T, Fujimura H (2018). A pilot study of tranilast for cardiomyopathy of muscular dystrophy. Intern Med.

[11] Iwata Y, Matsumura T (2019). Blockade of TRPV2 is a novel therapy for cardiomyopathy in muscular dystrophy. Int J Mol Sci.

[12] Matsumura T, Hashimoto H, Sekimizu M, Saito AM, Iwata Y, Asakura M (2021). Study protocol for a multicenter, open-label, single-arm study of tranilast for cardiomyopathy of muscular dystrophy. Kurume Med J.

[13] Demachi J, Kagaya Y, Watanabe J, Sakuma M, Ikeda J, Kakuta Y (2004). Characteristics of the increase in plasma brain natriuretic peptide level in left ventricular systolic dysfunction, associated with muscular dystrophy in comparison with idiopathic dilated cardiomyopathy. Neuromuscul Disord.

[14] Takekoshi N, Kanemitsu S, Kitayama M, Masuyama K, Kanaya H, Namura M (1996). Percutaneous transluminal coronary angioplasty (PTCA) -a phase III multicenter open trial. Kiso Rinsho.

[15] Kato K, Tamai H, Hayakawa H, Yamaguchi T, Kanmatsuse K, Haze K (1996). Clinical evaluation of tranilast on restenosis after percutaneous transluminal coronary angiopathy (PTCA)—a double-blind placebo-controlled comparative study. Rinsho Iyaku.

[16] Matsumura T, Saito T, Fujimura H, Shinno S (2007). Cardiac troponin I for accurate evaluation of cardiac status in myopathic patients. Brain Dev.

[17] Matsumura T, Takada H, Kobayashi M, Nakajima T, Ogata K, Nakamura A (2021). A Web-based questionnaire survey on the influence of coronavirus disease-19 on the care of patients with muscular dystrophy. Neuromuscul Disord.

[18] Matsumura T, Tamura T, Kuru S, Kikuchi Y, Kawai M (2010). Carvedilol can prevent cardiac events in Duchenne muscular dystrophy. Intern Med.

[19] Madokoro N, Ito Y (2013). The occupational therapy effect for Duchenne muscular dystrophy patients by the randomized clinical trial. J Jpn Health Sci.

